# The impact of biopsy core length on the discrepancy in Gleason scores between biopsy and radical prostatectomy specimen

**DOI:** 10.1002/bco2.70009

**Published:** 2025-03-04

**Authors:** Cheng‐hao Guo, Yin‐shuai Geng, Liang‐yong Zhu, Xue‐fei Ding, Yang Luan

**Affiliations:** ^1^ Medical School of Yangzhou University Yangzhou Jiangsu Province China; ^2^ Department of Urology Ansteel General Hospital Anshan Liaoning Province China; ^3^ Department of Urology Northern Jiangsu People's Hospital Affiliated to Yangzhou University Yangzhou Jiangsu Province China

**Keywords:** Gleason score discrepancy, prostate cancer, radical prostatectomy, transperineal prostate biopsy

## Abstract

**Objective:**

To assess the impact of the biopsy core length on the discrepancy in Gleason score between biopsy and radical prostatectomy specimens.

**Patients and Methods:**

Retrospective analysis of clinical data from 247 patients who underwent transperineal prostate biopsy and radical prostatectomy of prostate cancer at our centre from 2022 to 2023. The clinical data included age, pre‐biopsy prostate‐specific antigen (PSA) levels, prostate volume, number of biopsy needles, number of positive biopsy needles, biopsy core length, biopsy Gleason score and post‐radical prostatectomy Gleason score. Statistics were analysed by SPSS26.

**Result:**

On histopathological examination, no changes in the Gleason score were observed in 127 (51.4%) patients, whereas the Gleason score was upgraded in 101 (40.9%) patients and downgraded in 19 (7.7%) patients at radical prostatectomy. Average biopsy core length for Gleason score upgraded on radical prostatectomy (44.3 %, n = 101) was 11.11 ± 1.34 mm compared to 11.88 ± 1.03 mm(p < 0.01)for Gleason score consistent(55.7 %, n = 127). The multivariate logistic regression analysis revealed a significant association between the biopsy core length (P < 0.01, OR = 0.556, 95%CI: 0.429–‐0.722) and prostate volume (P < 0.05, OR = 0.982, 95%CI: 0.429–‐0.722), with both factors being significantly correlated with radical prostatectomy Gleason score increase. Furthermore, these variables were identified as independent predictors of radical prostatectomy Gleason score increase and exhibited a negative correlation. The biopsy core length was evaluated using a receiver operating characteristic (ROC) curve, with a cutoff value of 11.4 mm for the accurate diagnosis of prostate cancer (AUC: 0.702, sensitivity: 75.6%, specificity 51.2%, P < 0.001).

**Conclusion:**

The concordance between biopsy and radical prostatectomy may be improved with a longer biopsy core length. To enhance the consistency of Gleason scores between biopsy and radical specimens, it is recommended that the biopsy core length be at least 11.4 mm. Patients with a smaller prostate volume are at a higher risk of experiencing discordant pathological grades between biopsy and radical prostatectomy.

## 1 INTRODUCTION

Prostate cancer is one of the most prevalent malignant tumours in clinical practice among males, and its global incidence has been gradually increasing in recent years.^1^ Transrectal ultrasound (TRUS)‐guided biopsy is the primary method for diagnosing prostate cancer in clinical practice. The Gleason score obtained from biopsy specimens serves as a fundamental tool for risk stratification, playing a crucial role in determining the clinical stage and subsequent treatment of patients. However, based on existing research results, there is often a significant discrepancy in Gleason score between biopsy specimens and radical prostatectomy specimens, with more than one‐third of patients experiencing an upgrade or downgrade in Gleason score after radical prostatectomy.^2^, ^3^, ^4^, ^5^ Misclassification of biopsy specimens may result in overtreatment or undertreatment of patients and have a strong influence on patients' long‐term prognosis.^6^ Therefore, it is essential to gain a better understanding of the discrepancies in the Gleason score between biopsy specimens and radical prostatectomy specimens to deal with such issues effectively.

The essence of prostate biopsy is a random sampling biopsy of the tumour. The Gleason score of the sample is affected by many factors such as the location of the tumour, the multifocality of the tumour, the omission of the tumour, the heterogeneity of the tumour and the pathological reading, which makes the biopsy specimen unable to comprehensively reflect the malignant degree of the disease. At present, the clinical research on the discrepancy of Gleason score mainly focuses on the clinical parameters that can be used as predictive factors. Currently, the known clinical parameters include age, PSA, prostate‐specific antigen density, body mass index, clinical tumour stage, number of biopsy needles, prostate volume, tumour tissue percentage and the interval between biopsy and radical prostatectomy.^5^, ^6^, ^7^, ^8^, ^9^

Although various factors can be utilized to predict the discrepancy in Gleason score between biopsy specimens and radical prostatectomy specimens, most of these factors are fixed variables that merely serve as guidance in clinical practice. It mainly serves to predict an upgrade in the Gleason score for patients after surgery. The quality of the biopsy tissue has been proven to hold significant value in prostate cancer diagnosis.^10^ However, there is a scarcity of clinical studies investigating the relationship between biopsy core length and Gleason score variation in radical prostatectomy specimens. Therefore, this retrospective study aims to analyse the clinical data of patients who underwent transperineal prostate biopsy and radical prostatectomy at our centre, with an objective to evaluate the impact of biopsy core length on the discrepancy between biopsy Gleason score and post‐radical prostatectomy Gleason score.

## 2 MATERIALS AND METHODS

### 2.1 General information

The clinical data were collected from 247 patients, who were diagnosed with prostate cancer through ultrasound‐guided transperineal prostate biopsy and subsequently underwent laparoscopic/robotic radical prostatectomy at Northern Jiangsu Hospital from 2022 to 2023. The collected data included age, pre‐biopsy PSA levels, number of biopsy needles, number of positive biopsies needles, biopsy core length, biopsy Gleason score and post‐radical prostatectomy Gleason score. Patients who had received chemotherapy, radiotherapy, or endocrine therapy prior to surgery were excluded from the study. All clinical data was obtained from our hospital's Health Information System.

### 2.2 Method of biopsy

**FIGURE 1:**
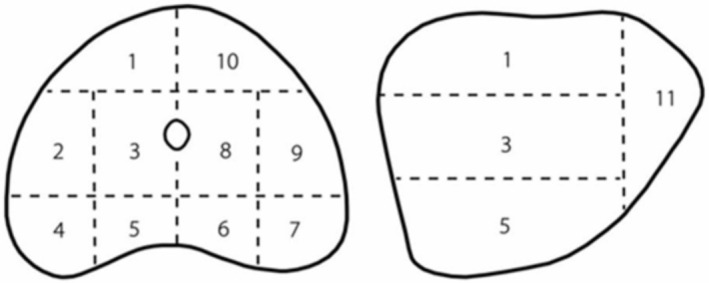
Prostate biopsy zones.

All biopsies were performed under lumbar or general anaesthesia. According to the anatomical characteristics of the prostate, the prostate was divided into two planes and 11 regions (Figure 1), of which zone 1–10 was the base of the prostate, and region 11 was the apex of the prostate, which were divided into 11–1, 11–2, 11–3 and 11–4 according to the order of upper right, lower right, lower left and upper left. The patient was placed in the supine position at the time of biopsy, and a rectal ultrasound probe (Flexfocus1202, ‐BK, Denmark) was fixed to the shelf and placed into the rectum. Under real‐time ultrasound guidance, in accordance with the volume of the prostate, 18G biopsy needles (MC1820, Bard, USA) were employed for insertion through the perineum, and 1 to 3 biopsies were conducted in each region of the prostate. During the biopsy procedure, if the patient's preoperative MRI or intraoperative ultrasound reveals abnormal signals, the surgeon will perform biopsy of this area and classify it according to the corresponding region. It is important to note that the presence of abnormal signals does not influence the biopsy method or the number of biopsy needles utilized in this study.

The specimens of each biopsy area were placed in a specimen bottle, and the tissue was removed from the biopsy needle in strict accordance with the principle of sterility and careful to avoid damage to the tissue strip. After operation, 4% formalin was injected into the specimen bottle and sent for examination.

### 2.3 Pathological data

**FIGURE 2:**
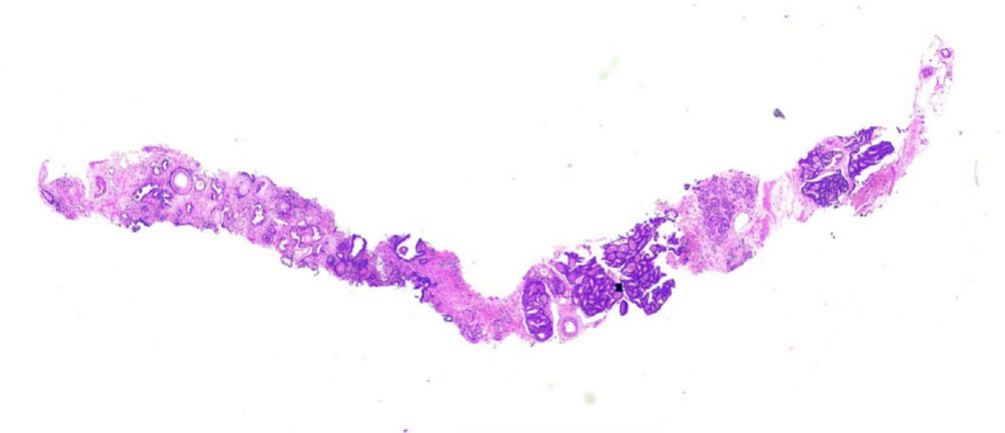
Biopsy tissue strip.

The biopsy sample sections from all included patients were collected, and the lengths of each needle core were measured and recorded in millimetres (mm) (Figure 2). In cases where multiple biopsy samples were taken from the same area, all samples were measured, and their average value was used to represent the biopsy needle core length in that region. Additionally, for regions 11.1, 11.2, 11.3 and 11.4, their lengths were averaged to determine the biopsy needle core length in this particular region.

The biopsy samples were evaluated by a pathologist specialized in diagnosing urinary tumour diseases at Northern Jiangsu People's Hospital, and the results for each region were scored according to the 2016 WHO Gleason score criteria for urinary male reproductive system tumours. Subsequently, a final biopsy Gleason score was determined using an overall scoring method based on these individual scores.^11^ To minimize potential diagnostic bias, the final pathological Gleason score obtained after radical prostatectomy was also scored by the same pathologist.

In this study, upgrading of Gleason score referred to an increase in radical prostatectomy Gleason score compared with biopsy Gleason score; whereas downgrading of Gleason score referred to a decrease in radical prostatectomy Gleason score compared with biopsy Gleason score.

### 2.4 Statistical methods

The data were analysed by SPSS26.0 statistical software, and the measurement data were expressed as mean ± standard deviation (x ®±s). T‐test and Mann–Whitney U test were used to compare the measurement data between groups with normal distribution and non‐normal distribution, respectively. Logistic regression analysis was used to calculate the risk factors of Gleason score change. A p‐value of 0.05 or less was considered statistically significant in this study.

## 3 RESULT

A total of 247 patients were finally included in our study. The average patient age was 70.29 ± 6.83 years (47–88). The average patient PSA was 21.31 ± 23.32 ng/ml (0.54–148). The average prostate volume of the radical prostatectomy specimens was 49.05 ± 31.24 ml (14.65–312.00). The median number of biopsy needles was 15.78 ± 1.91(14–22). The median number of positive biopsy needles was 2.51 ± 2.09(1–11).

In the needle biopsies, the percentage of Gleason score 3 + 3, 3 + 4, 4 + 3,8,≥9 occurred in 38.9% (96/247), 23.5% (58/247),14.2% (35/247), 10.5% (26/247) and 12.9% (32/247), respectively. In the final histopathological, the percentage of Gleason score 3 + 3, 3 + 4, 4 + 3,8, ≥ 9 occurred in 19.8% (49/247), 30.8% (76/247), 14.6% (36/247), 13.8% (34/247) and 21% (52/247). (Table 1).

**TABLE 1:** Patient's characteristics and results.

Patients	‐	n = 247
Age(X ± SD), years	‐	70.29 ± 6.83
PSA(X ± SD), ng/ml	‐	21.31 ± 23.32
Prostate volumes(X ± SD), ml	‐	49.05 ± 31.24
Number of biospy cores(X ± SD), n	‐	15.78 ± 1.91
Number of positive biospy cores(X ± SD), n	‐	2.51 ± 2.09
Core length(X ± SD), mm	‐	11.6 ± 1.3
Biopsies Gleason score (%)	3 + 3 3 + 4 4 + 3 8 9–10	96, (38.9%) 58, (23.5%) 35, (14.2%) 26, (10.5%) 32, (12.9%)
Specimens (Gleason)	3 + 3 3 + 4 4 + 3 8 9–10	49, (19.8%) 76, (30.8%) 36, (14.6%) 34, (13.8%) 52, (21.0%)

After histopathological examination, the proportion of Gleason score consistent group was 127 (51.4%) patients, the proportion of Gleason score upgrade group was 120 (48.6%), include upgraded in 101 (40.9%) patients and downgraded in 19 (7.7%) patients. The upgrade rate of biopsy Gleason score 3 + 3, 3 + 4, 4 + 3,8, occurred in 51.0% (49/96), 43.1% (25/58), 57.1% (20/35), 26.9% (7/26), respectively. In patients with Gleason score 3 + 3, the upgrade to 3 + 4, 4 + 3, 8, 9–10 were 35 (36.5%), 7 (7.3%), 6 (6.2%), 1 (1.0%), respectively (Table 2).

**TABLE 2:** The Gleason score change after radical prostatectomy.

	Radical prostatectomy gs						
Biopsy GS	‐‐‐‐‐	Gs 3 + 3	Gs 3 + 4	Gs 4 + 3	Gs 8	Gs9、10	TOTAL
	Gs 3 + 3	47	35	7	6	1	96
	Gs 3 + 4	2	31	14	6	5	58
	Gs 4 + 3	0	5	10	10	10	35
	Gs 8	0	4	5	10	7	26
	Gs9、10	0	1	0	2	29	32
	TOTAL	49	76	36	34	52	247

According to the biopsy pathological results and radical prostatectomy pathological results, the patients were divided into Gleason score consistent group and Gleason score upgraded group, of which 127 patients were included in Gleason score consistent group and 101 patients were included in Gleason score upgraded group. There were no significant discrepancies in age, pre‐biopsy PSA levels, number of biopsy needles, number of positive biopsies needles between the two groups. However, the average prostate volume of the Gleason score upgraded group was significantly lower than that of the Gleason score consistent group (43.01 ± 20.20 ml vs 53.07 ± 34.44 ml, p < 0.01). The average biopsy core length of Gleason score upgraded group was significantly shorter than that of Gleason score consistent group (11.11 ± 1.34 mm VS 11.88 ± 1.03 mm, p < 0.01) (Table 3).

**TABLE 3:** A comparative analysis between the upgraded and the consistent groups.

Parameters	Total	GS consistent	GS upgrade	P‐VALUE
Number(%)	228(100)	127(55.7)	101(44.3)	‐
Age(X ± SD), years	70.12 ± 6.97	69.69 ± 7.42	70.66 ± 6.36	0.289
PSA(X ± SD), ng/ml	21.17 ± 23.43	21.35 ± 23.19	20.93 ± 23.85	0.696
Prostate volumes(X ± SD), ml	48.62 ± 29.38	53.07 ± 34.44	43.01 ± 20.20	0.008
Number of biospy cores(X ± SD), n	15.77 ± 1.88	15.94 ± 1.97	15.55 ± 1.75	0.149
Number of positive biospy cores(X ± SD), n	2.25 ± 2.03	2.39 ± 1.81	2.65 ± 2.28	0.768
Core length(X ± SD), mm	11.54 ± 1.24	11.88 ± 1.03	11.11 ± 1.34	P < 0.01

Among the 247 enrolled patients, 159 (64.4%) underwent prostate MRI prior to biopsy, while 88 (35.6%) did not. Among the patients who received an MRI before biopsy, 39.6% (63/159) exhibited upgrading after radical prostatectomy, and for those who did not undergo MRI, 43.2% (38/88) presented upgrading after radical prostatectomy. The difference in the probability of upgrading between the two was not statistically significant.

In univariate analysis, the mean core length and the mean prostate volume were associated with gleason upgrading on radical prostatectomy (p < 0.05). In addition, we used p < 0.2 as the inclusion criterion for multivariate logistic regression analysis to include the average number of biopsy needles in the multivariate analysis (P = 0.149).

In multivariate analysis, the average core length and the average prostate volume were protective factors for biopsy Gleason score upgrade (P < 0.05 or) P < 0.01).

The biopsy core length was evaluated using a ROC curve, with a cutoff value of 11.4 mm for the accurate diagnosis of prostate cancer (AUC: 0.702, sensitivity: 75.6%, specificity 51.2%, P < 0.001).

## 4 DISCUSSION

Although various factors have been confirmed to influence the discrepancy of Gleason score in clinical practice, it is important to note that the results regarding these influencing factors are not consistent and some remain controversial. Additionally, most of these factors are fixed variables, mainly serve a predictive function in clinical practice and contribute to improving biopsy strategies, thereby reducing the occurrence of Gleason score discrepancy. However, it should be noted that this approach may face limitations in terms of widespread implementation across multiple centres due to the lack of standardized criteria. Prostate biopsy serves as a crucial foundation for prostate cancer diagnosis, the biopsy core length also playing a pivotal role in pathologists' assessment of tumour grade.^10^ In comparison to predicting and intervening on Gleason score discrepancy through various factors, a simpler approach would involve directly ensuring or increasing biopsy core length by improving the biopsy process.

Gleason score, which combines the major and minor Gleason score, is utilized to categorize prostate cancer into five prognostic grade groups. Accurate determination of the clinical stage of prostate cancer and development of an appropriate treatment plan heavily rely on the Gleason score obtained from prostate biopsy, as it is closely associated with prognosis, recurrence and even mortality.^12^, ^13^ Currently, there are variations in reported Gleason score for biopsy cores in clinical practice. The commonly employed scoring methods include: 1) highest score (based on the highest Gleason score regardless of proportion), 2) overall score (based on a combination of all Gleason score) and 3) largest volume score (based on the Gleason score of the lesion with the largest volume).^14^ Historically, European and American countries have predominantly utilized the highest score method.^15^ Whereas the research findings in recent years have indicated that the adoption of the overall scores can likewise offer a superior prediction of the patients' outcomes, and the performance of the overall score in the face of the gleason 3 + 4 and gleason 4 + 3 subgroups is significantly superior to that of the highest score.^11^ Therefore, this study employed an overall score approach for biopsy needles.

The length and quality of biopsy cores have been demonstrated to significantly impact the detection rate of prostate cancer.^10^, ^16^, ^17^ In the present study, it was also observed that core length influenced the Gleason score discrepancy between biopsy specimens and radical prostatectomy specimens. Despite no significant variation in the number of biopsy needles, the biopsy core length in the Gleason score upgraded group was significantly lower compared to that in the Gleason score consistent group (11.11 ± 1.34 mm vs 11.88 ± 1.03 mm, p < 0.01). The present study also hypothesize that this could be attributed to longer core lengths yielding more effective tumour tissues and providing crucial pathological information, thereby enhancing Gleason score consistency ‐ similar to how longer core length are associated with higher tumour detection rates. Additionally, the research findings of Xuefei D et al.^18^ and Choi et al.^19^ demonstrate that thicker biopsy needles, along with an increase in the number of biopsy needles and biopsy density, can all obtain more effective tumour tissues, thereby enhancing the detection efficiency of prostate cancer. Although the aforementioned studies did not specifically examine consistency between core lengths and Gleason score, these aforementioned research results provide strong support for our study by highlighting how improving biopsy tissue volume (thickness, number) aids in diagnosing prostate cancer.

In prior research on biopsy core length for the diagnosis of prostate cancer, various lengths ranging from 10 mm to 12 mm have been suggested to ensure effective diagnosis.^16^, ^20^, ^21^ In our study, the optimal cut‐off value for biopsy core length, as determined by the ROC curve, is 11.4 mm, aligning closely with the findings of previous studies. It is important to note, however, that the cut‐off value established in this study primarily targets the consistency of Gleason score between biopsy and radical prostatectomy specimens, rather than the detection rate of prostate cancer. This suggests that a biopsy core length of 11.4 mm or greater may concurrently achieve a satisfactory detection rate for prostate cancer and a high degree of consistency in Gleason score between biopsy and radical prostatectomy specimens. Consequently, we recommend that during transperineal prostate biopsy, the biopsy core length should not fall below 11.4 mm; if the length is shorter, repeat biopsy in the corresponding region are advised. In clinical practice, the length of the biopsy core is influenced by multiple factors, with transperineal biopsy being considered more likely to obtain high‐quality prostate tissue compared to transrectal biopsy.^22^ Factors such as the proficiency of the operator and biopsy habits also significantly impact the length of core.^23^ Additionally, different regions of the prostate, as well as the type and model of the biopsy needle affect both the length and integrity of tissue cores.

The study also discovered that Gleason score upgrades were predominantly observed in patients with a biopsy score of Gleason score 3 + 3, accounting for 48.5% (49/101) of all patients with an upgrade; furthermore, 51% of patients with a biopsy score of Gleason score 3 + 3 experienced an upgrade after radical prostatectomy, including 35 cases upgraded to Gleason score 3 + 4 and 7 cases upgraded to Gleason score 4 + 3. These findings are consistent with the research by Zhuo et al^24^ on the frequency of upgrading in low‐risk patients following surgery, which indicates that more than half of patients with a biopsy Gleason score 6 have undetected higher‐grade lesions. This poses significant challenges for the development of subsequent treatment plans. However, among patients with a biopsy score of Gleason score 3 + 4,24.1% (14/58) were upgraded to Gleason score 4 + 3, and among those initially scored as Gleason score 4 + 3,14.2% (5/35) were downgraded to Gleason score 3 + 4.These results indicate inaccuracies in assessing the proportion of gleason 4 tumours using only biopsy scores. Considering that the upgrade of Gleason scores has a significant impact on the subsequent treatment plans for patients with Gleason score 6–7 subgroups, we plan to conduct more in‐depth research into the risk factors associated with post‐radical prostatectomy Gleason score upgrading. This research aims to enhance the accuracy of risk stratification for these patients.

In our study, only patients who underwent systematic biopsy were included. Despite over 60% of the patients undergoing prostate MRI prior to biopsy, many did not proceed with MRI‐targeted prostate biopsy due to economic constraints and the lack of clear target signals on MRI. Consequently, we were unable to assess the effect of MRI‐targeted biopsy on detection accuracy, which represents a limitation of this study. According to Diamand et al.,^25^ the consistency between systematic biopsy and targeted biopsy with the postoperative Gleason score after radical prostatectomy is relatively comparable, at 49.4% versus 51.2%. And Stefano et al.^26^ reported no statistically significant difference in the consistency between systematic biopsy and targeted biopsy with the postoperative Gleason score after radical prostatectomy when detecting International Society of Urological Pathology Grade Group 1 (ISUP GG1) cases. Given that systematic biopsy is far more commonly performed than targeted biopsy in clinical practice, this underscores the practical relevance and generalizability of our findings. Furthermore, irrespective of whether patients underwent MRI examination prior to biopsy, all patients in this study were diagnosed via transperineal biopsy. This approach may influence the detection outcomes to some extent. Although Hu et al.^27^ found that under standardized prostate MRI protocols, transperineal and transrectal biopsies yield comparable detection rates for clinically significant prostate cancer, it must be acknowledged that for tumours located in specific anatomical regions, transrectal biopsy may offer advantages over transperineal biopsy.

Regarding other factors that predict Gleason score upgrade, our study also identified that prostate volume is inversely associated with postoperative Gleason score upgrade. Specifically, a smaller prostate volume correlates with a higher likelihood of postoperative Gleason score upgrade. This finding aligns with the results reported by Epstein et al^6^ concerning prostate volume. We deemed that this association might be attributed to the delayed onset of symptoms in smaller prostates, leading to advanced disease progression at the time of diagnosis, as well as fewer biopsy needles obtained from smaller prostates. Furthermore, in our study, the relationship between age, PSA and Gleason score upgrading was not statistically significant. By comparing studies on other age groups and PSA as a risk factor for Gleason score upgrading,^5^, ^6^, ^8^ it is found that on the one hand, the number of patients included in our study was a relatively small sample cohort; on the other hand, our patient cohort exhibited a broad distribution of age and PSA levels without stratification, which might have influenced the final results. Furthermore, other studies have reported that f‐PSA and PSA density are associated with postoperative Gleason score upgrading,^28^ suggesting a potentially more intricate relationship between PSA and prostate volume in predicting Gleason score upgrades. Since the primary focus of this study was on the correlation between biopsy core length and postoperative Gleason score discrepancy, we did not explore this phenomenon further.

This study also has many shortcomings. Firstly, this study was a single‐centre retrospective investigation that may be subject to selection bias. Secondly, the relatively limited sample size does not preclude the potential for structural bias within the data and the enrolled cases. Thirdly, it should be noted that the measured length of the cores in this study refers to the length after embedding, fixation and sectioning, potentially leading to lower measurements than immediately after biopsy. Fourthly, the separate calculation of apex and basal tissue core lengths was not performed in this study, which could have an impact on the final results. Fifthly, further analysis was not conducted on patients who experienced Gleason score downgrading after radical prostatectomy due to the small sample size.

## 5 CONCLUSION

Our findings are demonstrated as follows: on the one hand, the biopsy core length significantly influences the disparity in the Gleason score following radical prostatectomy, with greater concordance observed as the biopsy core length increases. To enhance the consistency of Gleason scores between biopsy and radical specimens, it is recommended that the biopsy core length be at least 11.4 mm. On the other hand, prostate volume emerges as a protective factor against discrepancies in Gleason score, with larger volumes associated with higher likelihoods of consistency between biopsy specimens and radical prostatectomy specimens.

## AUTHOR CONTRIBUTIONS

We thank all the authors for participating in this study (Cheng‐hao Guo: wrote the paper and data analysis; Xue‐fei Ding: writing assistance and finance support; Yin‐shuai Geng: supply of materials; Liang‐yong Zhu: data analysis; Yang Luan: literature searching).

## CONFLICT OF INTEREST STATEMENT

The authors declare no conflicts of interest.
